# Human CIK Cells Loaded with Au Nanorods as a Theranostic Platform for Targeted Photoacoustic Imaging and Enhanced Immunotherapy and Photothermal Therapy

**DOI:** 10.1186/s11671-016-1468-8

**Published:** 2016-06-06

**Authors:** Yao Yang, Jingjing Zhang, Fangfang Xia, Chunlei Zhang, Qirong Qian, Xiao Zhi, Caixia Yue, Rongjin Sun, Shangli Cheng, Shan Fang, Weilin Jin, Yuming Yang, Daxiang Cui

**Affiliations:** Key Laboratory of Laboratory Medicine, Ministry of Education of China, Zhejiang Provincial Key Laboratory of Medical Genetics, School of Laboratory Medicine and Life Science, Wenzhou Medical University, Wenzhou, Zhejiang 325035 PR China; Institute of Nano Biomedicine and Engineering, Key Laboratory for Thin Film and Microfabrication Technology of the Ministry of Education, Department of Instrument Science and Engineering, School of Electronic Information and Electrical Engineering, Shanghai Jiao Tong University, 800 Dongchuan RD, Shanghai, 200240 PR China; Department of Surgery, Changzheng Hospital affiliated to Second Military Medical University, 151 Fengyang Road, Shanghai, 20003 PR China; National Center for Translational Medicine, Collaborative Innovational Center for System Biology, Shanghai Jiao Tong University, 800 Dongchuan Road, Shanghai, 200240 PR China

**Keywords:** Human CIK cell, Gold nanorods, Gastric cancer cell, Immunotherapy, Photothermal therapy, Photoacoustic imaging

## Abstract

How to realize targeted photoacoustic imaging, enhanced immunotherapy, and photothermal therapy of gastric cancer has become a great challenge. Herein, we reported for the first time that human cytokine-induced killer cells (CIK) loaded with gold nanorods were used for targeted photoacoustic imaging, enhanced immunotherapy, and photothermal therapy of gastric cancer. Silica-modified gold nanorods were prepared; then incubated with human cytokine-induced killer cells (CIK), resultant human CIK cells loaded with Au nanorods were evaluated for their cytotoxicity, targeted ability of gastric cancer in vitro and in vivo, immunotherapy, and photothermal therapy efficacy. In vitro cell experiment shows that human CIK cells labeled with gold nanorods actively target gastric cancer MGC803 cells, inhibit growth of MGC803 cells by inducing cell apoptosis, and kill MGC803 cells under low power density near-infrared (NIR) laser treatment (808-nm continuous wave laser, 1.5 W/cm^2^, 3 min). In vivo experiment results showed that human CIK cells labeled with gold nanorods could target actively and image subcutaneous gastric cancer vessels via photoacoustic imaging at 4 h post-injection, could enhance immunotherapy efficacy by up-regulating cytokines such as IL-1, IL-12, IL-2, IL-4, IL-17, and IFN-γ, and kill gastric cancer tissues by photothermal therapy via direct injection into tumor site under near-infrared (NIR) laser irradiation. High-performance human CIK cells labeled with Au nanorods are a good novel theranostic platform to exhibit great potential in applications such as tumor-targeted photoacoustic imaging, enhanced immunotherapy, and photothermal therapy in the near future.

## Background

Gastric cancer is the fourth commonest cancer and the second leading cause of cancer-related death worldwide [1–3]. It ranks number two among all malignant tumors in China according to the latest cancer disease spectrum [4]. The gastric cancer patient prognosis is very poor with 5-year survivals below 24 % [5]. How to cure completely gastric cancer has become a great challenge.

Cellular immunotherapy has become a promising therapeutic method to fight against cancers and is classified as class III medical treatment technology since 2009 in China [6–9]. Among various cellular immunotherapy methods, lymph cytokine activated killer (LAK) cells were firstly used for anti-cancer therapy by Elizabeth A. Grimm [10]. Then, Joseph H. Phillips finished a further research of the oncolytic effects executed by LAK cells. A kind of more powerful anti-tumor cell–tumor-infiltrating lymphocytes (TIL) were reported in 1986. After 5 years, Schmidt-Wolf invented a new culture method of cytokine-induced killer (CIK) cells—a better cellular immunotherapy than LAK cells and TIL. They kill tumor cells mainly via CD3^+^CD56^+^ cell mass [10–14]. Current reports show that CIK cells own the ability to target in vivo tumor cells [15, 16], homing to the tumor site through vascular perfusion. And the targeting characteristic of CIK cells has extensive adaptability feature, which may be applied to clinical tumor therapy. However, up to date, few reports have demonstrated that tumor patients with middle and late stages can be cured completely by using DCs and CIK immunotherapies. How to enhance CIK therapeutic effects has become a challengeable problem. Therefore, it is very necessary to develop new therapeutic strategies that could enhance CIK therapeutic effects and/or improve synergistic combined therapy.

Up to date, gold nanoparticle-based theranostics has achieved great advances for cancer prevention, imaging, and therapy [17–20]. For example, gold nanorods (GNRs) have caused more attention due to their unique optical properties and the potential applications. So far, various shapes of gold nanoparticles have been prepared, such as gold nanospheres, gold nanorods, gold nanoprisms (Au NPrs), gold nanostars, gold nanoshells, and gold nanowires [21–23], which strongly decide their different applications such as photothermal therapy (PTT) [24–26], photoacoustic imaging [27–29], and surface-enhanced resonance spectroscopy (SERS) [30]. Especially, gold nanorods have a relative bigger specific surface area to display an obvious surface plasmon resonance (SPR) band in the near-infrared (NIR) region of the electromagnetic (EM) spectrum and can be excited to produce heat efficiently [31, 32]. More important, the NIR radiation has a deeper penetration depth in biological tissues, which makes it an excellent theranostic agent for medical applications such as photoacoustic imaging and photothermal therapy (PTT) [33–35]. In our previous work, we realized controlled synthesis of gold nanorods and used gold nanorods to realize tumor targeting imaging, enhanced radiotherapeutic efficacy, and photothermal therapy [36, 37]. Due to gold nanorods’ good biocompatibility, we consider that gold nanorods have their own clinical translation prospect [18].

**Scheme 1:**
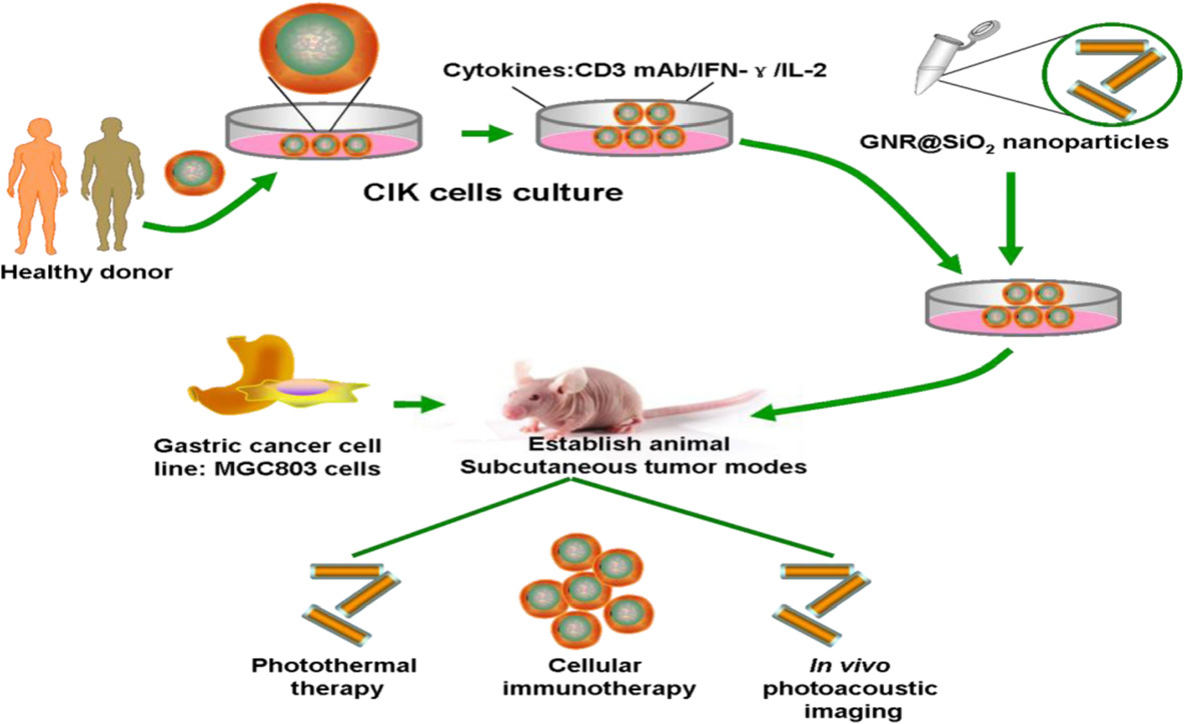
Schematic Illustration of preparation of human CIK cells loaded with gold nanorods and their applications in vivo

In this study, we fully take the advantages of human CIK cells and gold nanorods, with the aim of enhancing synergistic CIK cell therapeutic effects combined with PTT of gold nanorods. as in Scheme 1, we prepared human CIK cells, synthesized silica-modified gold nanorods, then prepared human CIK cells loaded with Au nanorods, and investigated the feasibility of human CIK cells loaded with Au nanorods for tumor targeting and PTT. Our results show that human CIK cells loaded with Au nanorods could target gastric cancer in vivo, realized photoacoustic imaging and enhanced immunotherapy, as well as photothermal therapy of gastric cancer, markedly enhanced CIK therapeutic efficacy. Our results provide a new strategy to treat gastric cancer, offer proof-of-principle of practicality of CIK combined with PTT based on human CIK cells loaded with gold nanorods, and exhibit potential clinical applications such as theranostics of gastric cancer and post-operation tumor patients and highly efficient delivery system for gene or drugs in near future.

## Methods

All animal experiments were approved by the Institutional Animal Care and Use Committee of Shanghai Jiao Tong University (SCXK-2012-0002).

### Material Source

Gold chloride trihydrate (HAuCl_4_ · 4H_2_O, 99 %), sodium borohydride (NaBH_4_), silver nitrate (AgNO_3_), L-ascorbic acid, and ethanol were obtained from Sinopharm Chemical Reagent Company (Shanghai, China). *N*-Cetyltrimethylammonium bromide (CTAB) were purchased from Sigma-Aldrich (St. Louis, MO, USA). Tetraethyl orthosilicate (TEOS) was purchased from Aladdin Reagent Co. Ltd (Shanghai, China). DiO perchlorate [3,3-dioctadecyloxacarbocyanine perchlorate], DiI iodide [1,1-dioctadecyl-3,3,3,3-tetramethylindocarbocyanine iodide], and DiR iodide [1,1-dioctadecyl-3,3,3,3-tetramethylindotricarbocyanine iodide] were obtained from AAT Bioquest Inc. (America). Annexin V-FITC/PI Apoptosis Detection Kit and Cell Counting Kit-8 (CCK-8) were purchased from Yeasen Corporation (Shanghai, China). MGC803 cell line and GES-1 cell line were available in the Cell Bank of Type Culture Collection of Chinese Academy of Sciences. Cytokine-induced killer cells were extracted from peripheral blood (PB) of healthy volunteers in our laboratory. Cell culture products and reagents, unless mentioned otherwise, were purchased from GIBCO. All the above chemicals were used without any further purification. Milli-Q water (18.2 MΩcm, Millipore Co., USA) was used in all the preparations. Serum-free X-VIVO15 medium was purchased from LONZA (USA). Recombinant human IL-2 (rhIL-2) was purchased from Invitrogen Corporation (GIBCO, USA). Anti-human CD3ε monoclonal antibody (CD3 mAb, OKT3) was purchased from Nobimpex (Germany). Recombinant human IFN-γ (rh IFN-γ) was obtained from Shanghai Clonbiotech Co., LTD (China).

### Synthesis and Characterization of Gold Nanorods

Gold nanorods were synthesized according to our previous report [22, 35]. The concrete steps are as follows: in brief, seed gold particles were formed by adding pre-iced aqueous NaBH_4_ (0.01 M, 600 μL) into the mix aqueous CTAB (0.1 M, 7.5 mL) and HAuCl_4_ (24.3 mM, 102.9 μL) rapidly while stirring. Then, the solution was stirred dramatically for 2 min and kept completely stationary at 30 °C for 2 h. Then, an aqueous solution (100 mL) of HAuCl4 (24.3 mM, 1.030 mL), AgNO3 (0.01 M, 1 mL), and CTAB (0.1 M, 100 mL) was treated with fresh ascorbic acid (0.1 M, 400 μL) and the solution changed color from bright yellow to colorless under vigorous stirring. The growth solution was then treated with a freshly prepared Au NP seed solution (3–5 nm; 0.24 ml) and began to turn red within 30 min. The end product was allowed to stand at room temperature overnight to yield a 100-mL suspension of GNRs with a longitudinal plasmon resonance (LPR) centered at 810 nm.

### Preparation and Characterization of Silica-Coated Au Nanorods

The prepared Au NRs (10 mL) were subjected to centrifugation at 10,000 rpm for 10 min and separated from the supernatant, then redispersed in Milli-Q water to a final volume of 10 mL. Then, 15 μL of 1 M NaOH solution was added under equably stirring to adjust the PH to 10.0. Following this step, 400 μL 10 mM TEOS in ethanol was added under gentle stirring per 30-min intervals (three times in total). The mixture was reacted for 24 h at 30 °C with gentle stirring. UV–vis spectra of Au NRs and Au NR@SiO_2_ nanoparticles were measured with a Varian Cary 50 spectrophotometer (Varian Inc., Palo Alto, CA, USA) equipped with a 10-mm quartz cell, where the light path length was 1 cm. The sizes and morphologies of the nanoparticles were characterized by high-resolution transmission electron microscope (HRTEM) on JEM-2100F (JEOL, Japan) with an acceleration voltage of 200 kV.

### Isolation, Culture, Expansion, and Phenotype Analysis of CIK Cells

By using a blood cell separator (Spectra v 6.1, Cobe, USA), (2–4) × 10^9^ PBMC cells from each healthy volunteer were obtained in a total volume of 50 mL. Cellular concentration was adjusted to 2 × 10^6^/mL in fresh serum-free X-VIVO 15 medium and incubated at 37 °C in a humidified atmosphere of 5 % CO_2_. To generate CIK cells, 2000 U/mL rh IFN-γ was added on the initial day. After 24 h of incubation, 50 ng/mL CD3 mAb and 1000 U/mL rhIL-2 were added. Fresh IL-2 and fresh X-VIVO 15 media were replenished every 3 days. On day 0, 4, 7, 10, 13, and 15, cell densities were determined. Incubated CIK cells (day 18) were collected, washed, and stained with anti-CD3-FITC and anti-CD56-PE antibodies (Becton Dickinson, USA). Cells were incubated with Abs for 30 min at 4 °C, and then the stained cells were washed and analyzed by BD FACSCalibur (BD Biosciences, Mountain View, CA). The CIK cells were collected on day 18 for DiR labeling and co-incubated with silica-modified Au nanorods.

### Cytotoxicity Analysis of Silica-Modified Au Nanorods by CCK-8 Assay

The MGC803 and GES-1 cells were maintained at 37 °C (5 % CO_2_) in Dulbecco’s Modified Eagle’s Medium (DMEM, HyClone) supplemented with 10 % (vol/vol) fetal bovine serum, 100 U mL^−1^ penicillin and 0.1 mg mL^−1^ streptomycin. Cell counting kit-8 (CCK-8) assay was carried out to investigate the toxicity of Au NR@SiO_2_ nanoparticles. In brief, MGC803 cells, GES-1 cells, and CIK cells were seeded in 96-well plate at a density of 5 × 10^3^ cells per well and cultured for 24 h. The cells in each well were incubated with 100-μL complete medium containing serial concentrations of Au NR@SiO_2_ nanoparticles (0~36 culture. After 24 h of incubation, 10 μL sterile CCK-8 per well was added and continued to incubate for 2 h at 37 °C (5 % CO_2_). The optical density values were determined at least in triplicate against a reagent blank at a test wavelength of 450 nm and reference wavelength of 630 nm.

Then, the human CIK cells labeled with silica-modified Au nanorods were collected and treated, the contents of Au in human CIK cells were measured by BIO-TEM (120-kV biology transmission electron microscope, FEI-Tecnai G2 spirit Biotwin, USA), and ICP-MS (inductively coupled plasma mass spectrometer, Agilent 7500a, USA).

### Photothermal Conversion Efficiency Measurement of Silica-Modified Au Nanorods

According to the result of ICP-MS, 6.788 M Au NR@SiO_2_ nanoparticles were redispersed in 1 mL final volume of X-VIVO medium, 1 Electron Milli-Q water respectively. Au NR@SiO_2_ nanoparticles in three solutions mentioned above were persistently excited by a 808-nm laser (1.5 W/cm^2^) for 4 min, and three solutions were treated as controls. According to this experiment, a heating curve can be determined.

### Effects of Silica-Modified Au Nanorods on Human CIK Cells

To observe the interaction between CIK and MGC803 cells, a Leica TCS SP8 cofocal laser scanning microscopy was applied to carry out in vitro confocal fluorescence imaging studies. DiO perchlorate and DiI iodide were used to label MGC803 and CIK cells, respectively. After labeling with cell membrane dyes, they were co-incubated for 12 h in the culture flask. DiO green fluorescence was excited at 488 nm with an argon ion laser, and the emission was collected from 500 to 510 nm. The red fluorescence of DiI was excited at 549 nm with an argon ion laser, and the emission was collected from 560 to 570 nm.

To investigate the effects of Au NR@SiO_2_ on the killing activity of CIK cells, CIK cells and CIK cells labeled with Au nanorods were respectively added into gastric cancer cell line (MGC803) and gastric mucosal epithelial cells (GES-1) at different ratios of effective cells to target cells (10~50:1) and co-incubated together for different hours. Then, the target cells were collected. An Annexin V-FITC/PI Apoptosis Detection Kit was used to measure the apoptotic and necrotic cells, according to the manufacturer’s protocol. The detection procedure worked as described below: After coincubation, the target cells were trypsinized, harvested, washed with 1× PBS, and resuspended in 200 μL of 1 × binding buffer containing 5 μL Annexin V-FITC and 10 μL PI. With incubation in dark at room temperature for 15 min, each sample was added 400 μL of binding buffer, and the cells were immediately analyzed by BD FACSCalibur (BD Biosciences, Mountain View, CA). The data analysis was performed with FlowJo 7.6 software. Positioning of quadrants on Annexin V/PI plots was performed to distinguish living cells (Annexin V −/PI −), early apoptotic cells (Annexin V +/PI −), late apoptotic cells (Annexin V +/PI +), and necrotic cells (Annexin V −/PI +).

### CIK Cells Labeled with Au Nanorods for Targeted Fluorescence Imaging

Firstly, gastric cancer-bearing nude mice models were prepared. Four weeks old female BALB/c nude mice (18–22 g) were obtained from Shanghai Slac Laboratory Animal Co. Ltd (Shanghai, China). Collected MGC803 cells were resuspended in 1× PBS, then 2 × 10^6^ cells/site was subcutaneously injected in the right flank of the mice. The tumors were allowed to grow for 3 weeks to reach a size of ≈150–200 mm^3^.

Fluorescent dye DiR was applied to label the cell membrane of human CIK cells labeled with Au nanorods. 5 × 10^6^ CIK cells labeled with Au nanorods were resuspended in 3-ml DiR working solution (concentration = 2.5 μM, fresh X-VIVO as solvent) then washed with 1× PBS twice after incubating at 37 °C (5 % CO_2_) for 20 min. The 5 × 10^6^ DiR-labeled CIK cells loaded with Au nanorods were resuspended in 100-μL 1× PBS and then were injected with multi-points around the tumor site. In vivo time-course fluorescence imaging (excitation 748/20 nm; emission 780/10 nm; exposure time 30 s) was performed by a Bruker In-Vivo F PRO imaging system (Billerica, MA, USA) at 0, 1, 3, 5, and 7 days. Then, at number 3 day post-injection of CIK cells, the mice sera in experiment group and control group were collected, and the cytokine levels such as IL-1, IL-12, IL-2, IL-4, IL-17, and IFN-γ were measured by commercial ELISA kit.

### CIK Cells Loaded with Au Nanorods for Targeted Photoacoustic Imaging

CIK cells loaded with Au nanorods were used for photoacoustic imaging of gastric cancer-bearing nude mice model. Gastric cancer-bearing nude mice were divided into three groups, control number 1 group (only injection of PBS), control number 2 group (only injection of Au nanorods (200 μg/mL)), experiment group (Au nanorods-labeled CIK, 1 × 10^6^ CIK cells incubated with 200 μg/mL for 24 h), which were respectively injected with PBS, Au nanorods, and Au nanorod-labeled CIK cells via tail veins into gastric cancer-bearing nude mice, and the images were collected at pre-injection, 6, 12, 24, and 72 h post-injection time point for excitation at 800 nm by NEXUS 128 photoacoustic imaging system.

### Human CIK Cells Loaded with Au Nanorods for Photothermal Therapy

1 × 10^6^ human CIK cells and CIK cells labeled with Au nanorods were respectively added into MGC803 gastric cancer cell line and gastric mucosal epithelial cells (GES-1) at different ratios of effective cells to target cells (10~50:1) and co-incubate for 24 h. The treatment group was exposed to a 808-nm laser (1.5 W/cm^2^) for 4 min. Then, the cell viability of control and treatment groups was analyzed by a Cell Counting Kit-8 kit.

Mice were divided into four groups (*n* = 5):1× PBS (100 NVivo cell and photothermal therapy, mice were divided into four groups (*n* = 5):by a cell counting ×10^6^ per tumor) resuspended in 100 μL 1× PBS were administered by peritumoral multi-point injection method; DiR-labeled CIK cells labeled with Au nanorods (5 × 10^6^ per tumor) resuspended in 100 μL 1× PBS were administered for two groups by peritumoral multi-point injection method (one for 808-nm laser irradiation for), only the mice in last group were exposed to a 808-nm laser (1.5 W/cm^2^, 7 days post-injection). The temperature changes of tumor site were recorded by an IR imaging sensor (Testo 875i, Germany).

### Biodistribution and Pathological Examination

MGC803 cells (5 × 10^6^) were injected subcutaneously into the right back flank area of 6- to 8-week-old female nude mice. Tumors were allowed to grow to a diameter of approximately 5 mm. At that point, 2 × 10^6^ CIK cells labeled with PEGylated Au nanorods were injected into the mice via the tail vein. Twelve mice were divided into four groups and then were sacrificed 1, 3, 5, and 7 weeks later. Several hundreds of microliter blood was immediately collected from the heart, mixed with 1 mg EDTA in microfuge tubes, and frozen in liquid nitrogen. Then, organs were collected and frozen in liquid nitrogen. Blood and tissue samples were completely lysed in aqua regia. Following with evaporation process, the resulting precipitates were dissolved in 0.5 M HCl. The Au content determination was conducted on an Agilent 7500a and every data point was expressed as a mean ± SD from triplicate samples. Data were represented as percentage of injection dose/organ.

The collected important organs were firstly fixed in 4 % neutral buffered formalin for 24 h, then dehydrated in 30 % sucrose solution, and processed into Tissue OCT-Freeze Medium and section. A standard H&E (hematoxylin and eosin) staining was performed to measure the histopathology changes of tumors.

### Statistical Analysis

All data were expressed as mean ± SD (standard error) of at least five independent experiments unless otherwise specified. Statistical analysis was performed by Student’s *t* test (*p* < 0.05) and was indicated to be statistical significant.

## Results and Discussion

### Preparation and Characterization of Au NRs and Au NR@SiO_2_

**Fig. 1:**
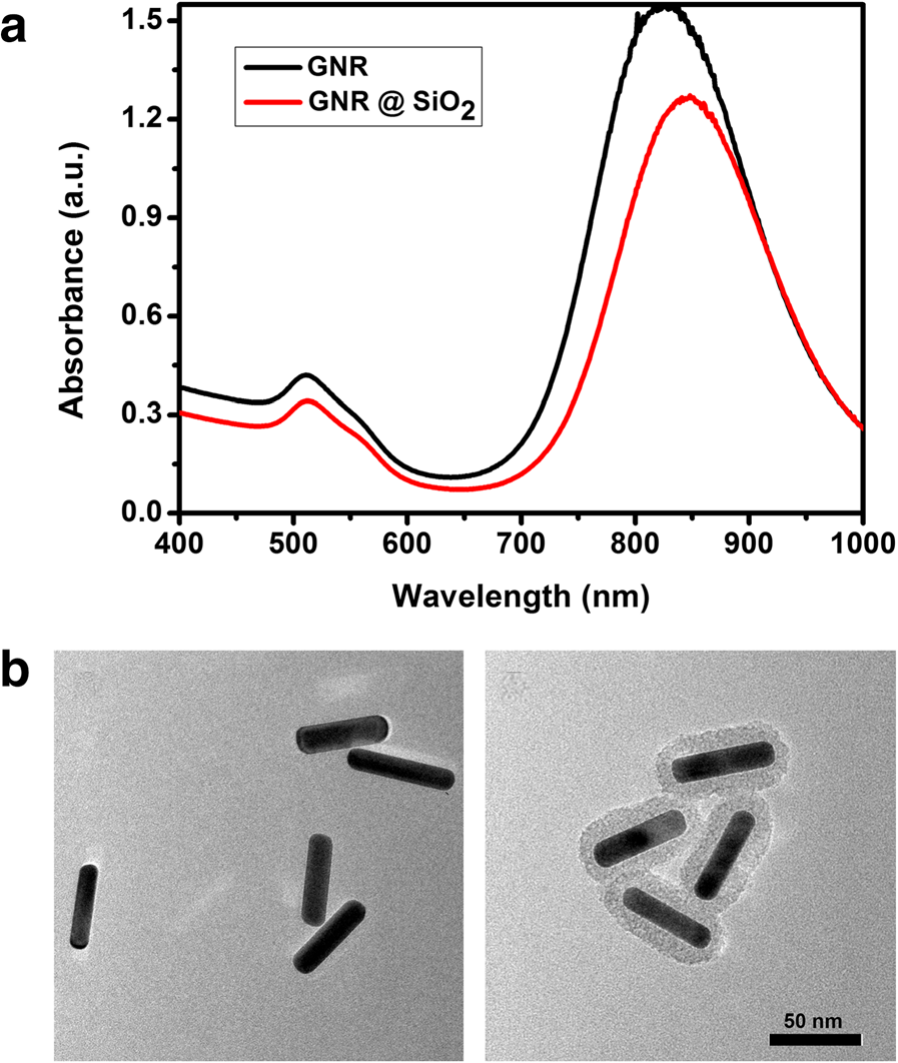
Characterization of the Au NR@SiO_2_ nanoparticles. **a** UV–vis spectra of Au NRs and Au NR@SiO_2_ nanoparticles. **b** HRTEM images of Au NRs and Au NR@SiO_2_ nanoparticles

Au nanorods were synthesized by seed-mediated growth method [34]; resultant CTAB-coated Au NRs were modified with SiO_2_. CTAB formed a bilayer around the Au NRs and served as an organic template for the formation of the silica layer on the surface of Au nanorods [36]. As shown in Fig. 1a, prepared Au nanorods have two absorption peaks, respectively located at 520 and 810 nm, and prepared Au NR@SiO_2_ nanoparticles have two absorption peaks, respectively located at 520 and 820 nm, exhibiting 10-nm red-shift. As shown in Fig. 1b, the synthesized Au nanorods have 60 nm in length and 8 nm in width; silica shell thickness was 20 nm or so in diameter.

### Preparation and Characterization of Human CIK Cells

**Fig. 2:**
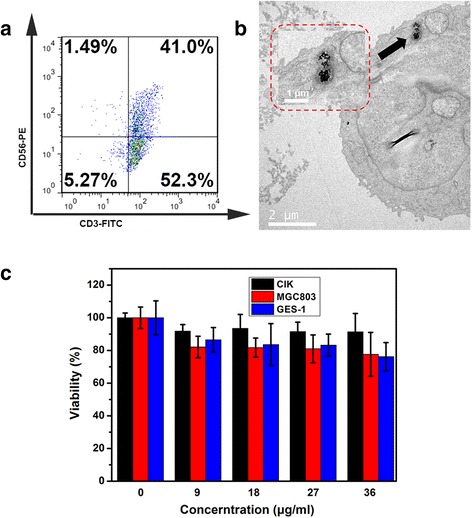
**a** Phenotype assay of cytokine-induced killer (CIK) cells: the percentage of CD3^+^CD56^+^ T cell is 41.0 % and CD3^+^ T cell represents more than 90 % of collected CIK cells. **b** Bio-TEM image of NP-CIK cell. **c** Cytotoxicity assay of several concentrations of Au NRs@SiO_2_ versus three kinds of cells—CIK, MGC803, and GES-1 cells

Cytokine-induced killer (CIK) cells are derived from peripheral blood mononuclear cells (PBMCs), which are usually obtained from the peripheral blood or cord blood. Three cytokines were added in the course of the cell culture: rh IFN-γ was used to induce some immune accessory molecules secretion and enhance the anti-tumor function of CIK cells [38]; CD3 monoclonal antibody was added into the culture liquid and induced higher level of cytotoxic activity and proliferation rate of human CIK cells [31]; added cytokine rhIL-2 can get a higher proliferation rate of CIK cells [32]. Schmidt-Wolf [13] et al. reported that the higher lytic activity of CIK cells as compared to LAK cells was mainly due to the higher proliferation of CD3^+^CD56^+^ T cells. CD3^+^CD56^+^ T cells were demonstrated to have non-MHC-restricted cytotoxicity and were very rare (1~5 %) in uncultured PBMCs. After co-culture with three kinds of cytokines for almost 3 weeks, as shown in Fig. 2a, the ratio of CD3^+^CD56^+^ T cells increased to 41 %, which highly suggest that human CIK cells including high concentration of CD3^+^CD56^+^ cell subpopulation were successfully prepared and owned a stronger anti-tumor bio-activity. As shown in Fig. 2b, prepared Au NR@SiO_2_ could have been gulfed into cytoplasm by human CIK cells. We also quantitatively determined the amount of Au NRs@SiO_2_ nanoparticles in CIK cells; the result showed that 13.576 μg Au existed in 1 × 10^7^ human CIK cells. As shown in Fig. 2c, prepared Au NR@SiO_2_ did not inhibit growth of CIK cells, MGC803 cells, and GES-1 cells, exhibiting low cytotoxicity, which fully demonstrate that Au NR@SiO_2_ NPs can label human CIK cells (NP-CIK cells), and owning good biocompatibility.

### Photothermal Conversion Efficiency of Au NRs@SiO_2_

**Fig. 3:**
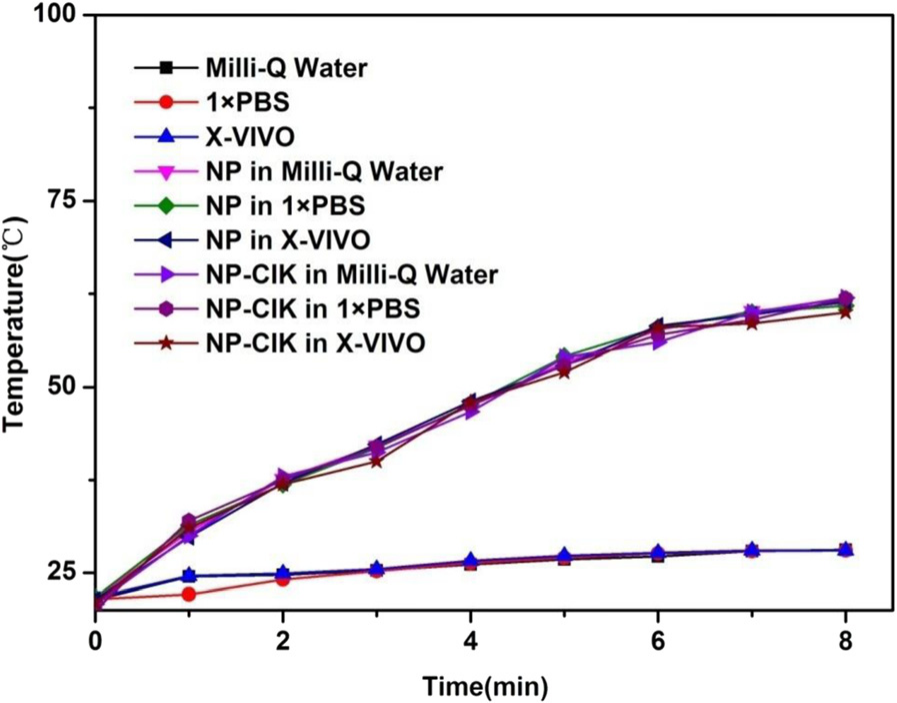
The heating curve of free Au NRs@SiO_2_ nanoparticles in three solvents

Au NRs@SiO_2_ nanoparticles have been broadly used for photothermal therapy of tumor [28, 35, 36]. But, few report is associated with human CIK cells labeled with Au NR@SiO_2_ (NP-CIK cells) for tumor target imaging and photothermal therapy. We measured the photothermal conversion efficiency of prepared Au NR@SiO_2_ nanoparticles under three kinds of different solvents such as Milli-Q water, 1× PBS, and X-VIVO medium; as shown in Fig. 3, three photothermal conversion efficiency curves of Au NRs@SiO_2_ nanoparticles were obtained (6.788 μg/ml per sample, 5 × 10^6^ CIK cells). The variation tendency of temperature is in accordance with the results of IR imaging (Fig. 6a). Free Au NRs@SiO_2_ nanoparticles can produce 60 °C under 808-nm NIR laser irradiation for 4 min. The temperature value is 55 °C at 2.5 min in heating curve and it is 57 °C in IR images at the same time point, which is enough for photothermal therapy. 5 × 10^6^ human CIK cells labeled with Au NR@SiO_2_ can produce the similar high temperature of 60 °C under the same condition.

### Effects of Human CIK Cells Labeled with Au NR@SiO_2_ on MGC803 Cells

Current studies show that CIK cells are a heterogeneous subset of ex vivo expanded T lymphocytes [10–12]. They present a mixed T-NK phenotype and are endowed with the MHC-unrestricted anti-tumor activity, which means it can kill cancer cells without attacking the normal cells [13, 14]. Some reports show that human CIK cells can target in vivo tumor cells. In the course of interaction between CIK cells and tumor cells, CIK cells exhibit killing tumor cell function. The anti-tumor mechanism of CIK cells was as follows: inducing target tumor cell apoptosis in the early stage and necrosis in the late stage [39]; released some chemical factors such as NKG2D, granzyme B, and perforin, killing tumor cells in the process of interaction between effective cells and target tumor cells [40].

**Fig. 4:**
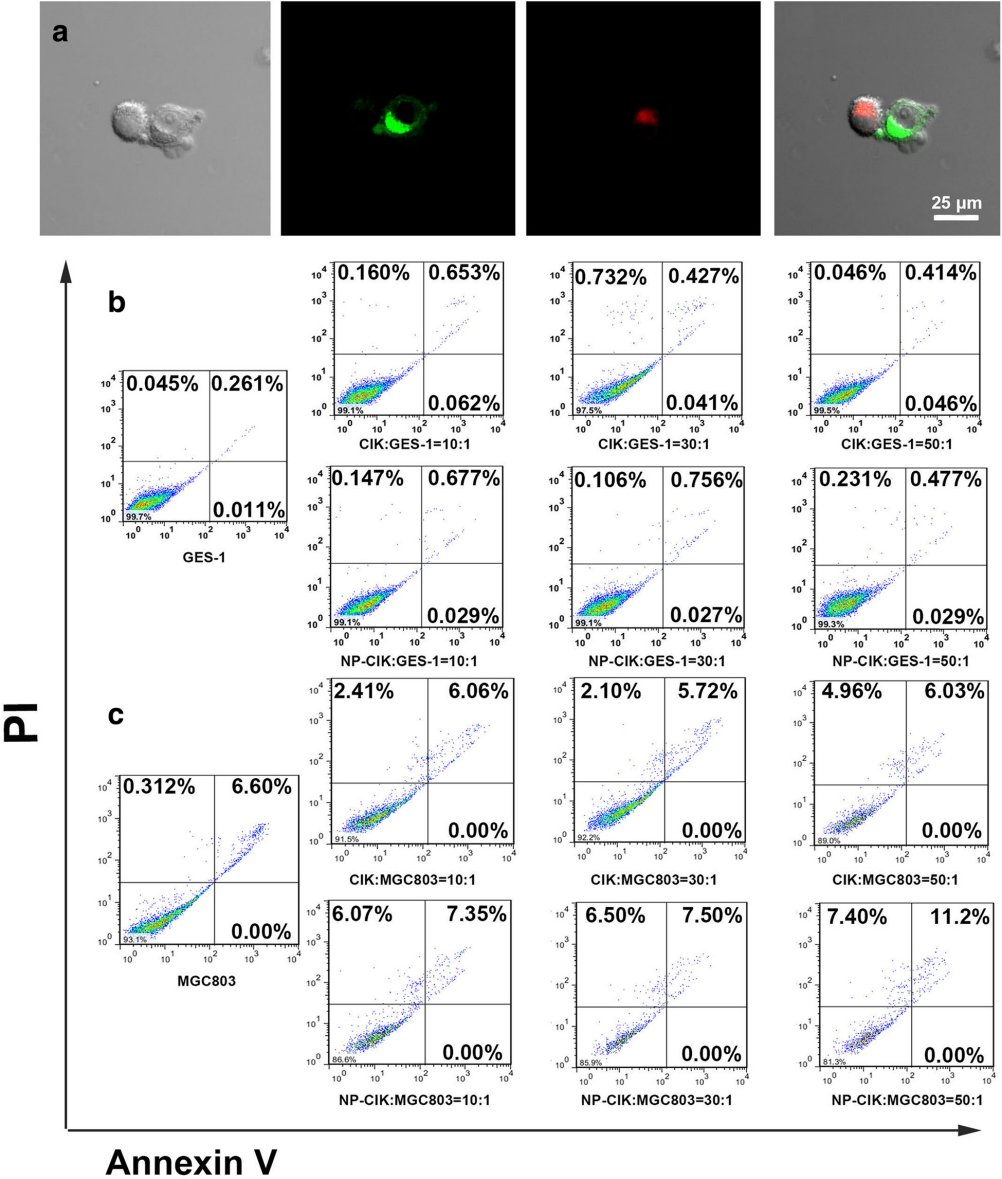
**a** Confocal images of the interactions between CIK cells (*red fluorescence*) and MGC803 cells (*green fluorescence*). **b**, **c** The apoptosis assay executed by flow cytometry

We also investigated the effects of human CIK cells labeled with Au NRs@SiO_2_ on gastric cancer MGC803 cells. With the aim of screening out the optimal ratio of CIK cells to gastric cancer cells, we added human CIK cells labeled with Au NR@SiO_2_ into MGC803 cells as the ratio of 10:1, 30:1, and 50:1 and then co-incubated for 12 h. Then, we used the confocal microscopy to observe cells. As shown in Fig. 4a, CIK cells actively target and home to the surface of MGC803 cells and then produce the anti-tumor function. As shown in Fig. 4b, human CIK cells cannot induce human normal gastric mucous cell GES-1 appear apoptosis, conversely, human CIK cells labeled with Au NRs@SiO_2_ can induce MGC803 cells appear apoptosis. Apoptosis cell number increased as the ratio of CIK cells to MGC803 cells increased. When the ratio of CIK cells to MGC803 cells reaches to 50:1, apoptosis cell number reaches to 11.2 %, which fully demonstrates that human CIK cells labeled with Au NR can kill gastric cancer cells via inducing cell apoptosis, and human CIK cell does not kill normal human gastric mucous cells [41, 42].

**Fig. 5:**
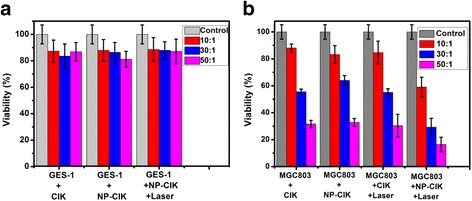
CCK-8 assay for CIK immunotherapy and photothermal therapy efficacy. **a** GES-1 cells were treated as the control group. **b** MGC803 cells were treated under four conditions

We also used CCK-8 kit to investigate the viability of MGC803 cells or GES-1 cells incubated with human CIK cells labeled with Au NR or only CIK cells. As shown in Fig. 5, human CIK cells and NP-CIK cells cannot inhibit growth of normal gastric mucous GES-1 cells but can inhibit the growth of gastric cancer MGC803 cells. As the ratio of human CIK cells to gastric cancer cells increased, the viabilities of gastric cancer MGC803 cells became lower and lower and was 32 %; under 1 W/cm^2^ 808-nm laser irradiation for 4 min, the viabilities of MGC803 cells became the lowest and was 18 %; there existed statistical difference between the test group of NP-CIK + laser irradiation for 4 min and the control group of only NP-CIK treatment, *P* < 0.01, which fully demonstrates that human CIK cells labeled with Au nanorod scan enhance immunotherapeutic efficacy of CIK cells under laser irradiation [43, 44].

### CIK Cells Labeled with Au Nanorods for Targeted Fluorescent Imaging

In order to investigate the feasibility of NP-CIK cells for targeted fluorescent imaging of gastric cancer in vivo, we prepared MGC803 gastric cancer-bearing nude mouse models, and 1 × 10^6^ NP-CIK cells were injected into nude mouse models via tail vein. At 24 h post-injection, 22 % of NP-CIK cells were located in gastric cancer site; as the post-injection day increased, the amount of NP-CIK cells in tumor site also increased; at 5 days post-injection, the amount of NP-CIK cells reached to 28 %, which show that NP-CIK cells can target in vivo gastric cancer cells.

**Fig. 6:**
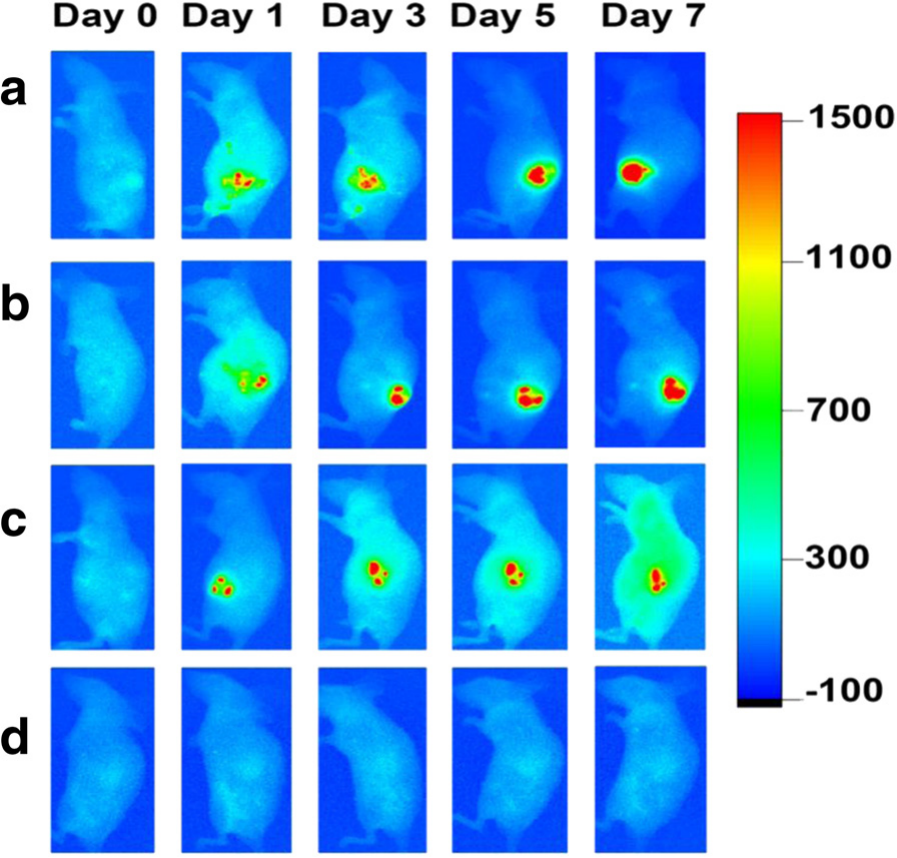
In vivo fluorescence imaging of NP-CIK and CIK cells. **a**, **b** NP-CIK cells injected. **c** CIK cells injected. **d** 1× PBS injected as control

In order to increase the amount of NP-CIK cells in the site of gastric cancer in vivo, 1 × 10^6^ NP-CIK cells labeled with DiR dye were injected into in vivo tumor site via peritumoral injection. As shown in Fig. 6, from 1 to 7 days post-injection, tumor sites always kept strong fluorescent signals, which show that DiR-labeled NP-CIK cells can stay in the tumor site for a long time. The amount of NP-CIK cells in tumor site via peritumoral injection is higher than the amount in tumor site via tail vein injection. NP-CIK cells enter into other organs such as the liver, spleen, lung, kidney, and brain, protect other important organs’ function, and own obvious advantages over tumor-targeted therapy based on NP-CIK cells via tail vein injection.

### NP-CIK Cells for Photoacoustic Imaging of Gastric Cancer In Vivo

**Fig. 7:**
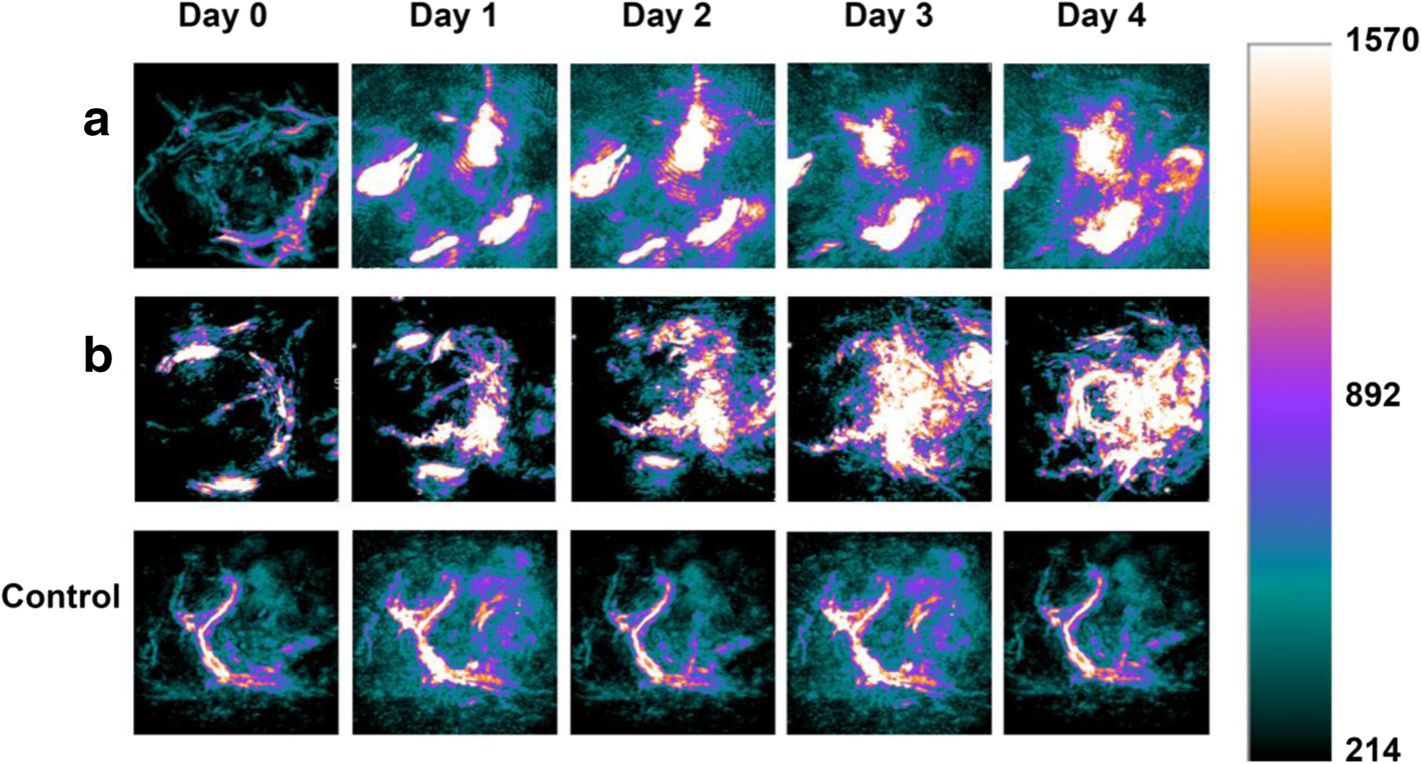
Photoacoustic (PA) images. **a** Au NR@SiO_2_ nanoparticles contained 6.788 μg Au resuspended in 100 μL 1× PBS via peritumor injection. **b** 5 × 10^6^ NP-CIK cells resuspended in 100 μL 1× PBS via peritumor injection (which contain 6.788 μg Au). (Control) 1× PBS (100 μL) injection as control. (day 0: before injection; day 1~4: four time points after injection)

The 720-nm NIR laser with moderate energy (~4 mJ per pulse) was used for photoacoustic imaging (PA) of the tumor in vivo, at which the wavelength neovascularization (CNV) was identified and the NP-CIK cells had a high PA contrast effect. As shown in Fig. 7, the PA signals of both NP (Fig. 7a) and NP-CIK cell (Fig. 7b) injection groups got a trend of continuous heightening. However, the intensity of PA signals in NP-CIK cell injection group (Fig. 7b) showed a more homogeneous effect than NP injection group (Fig. 7a). From this result, we can make a conclusion that the NP-CIK cells have a stronger ability of targeting and diffusion than simple NP injection. Basing on the information acquired from the PA images, the difference in the two test groups maybe due to the active targeting of CIK cells. Although the Au NR@SiO_2_ NPs also have the function to target the tumor site via the enhanced permeability and retention (EPR) effect [43, 44], the simple nanoparticles cannot obtain a higher efficiency of targeting than NP-CIK cell complex peritumor injection.

### In Vivo Biodistribution of CIK Cells Labeled with Au NRs

**Fig. 8:**
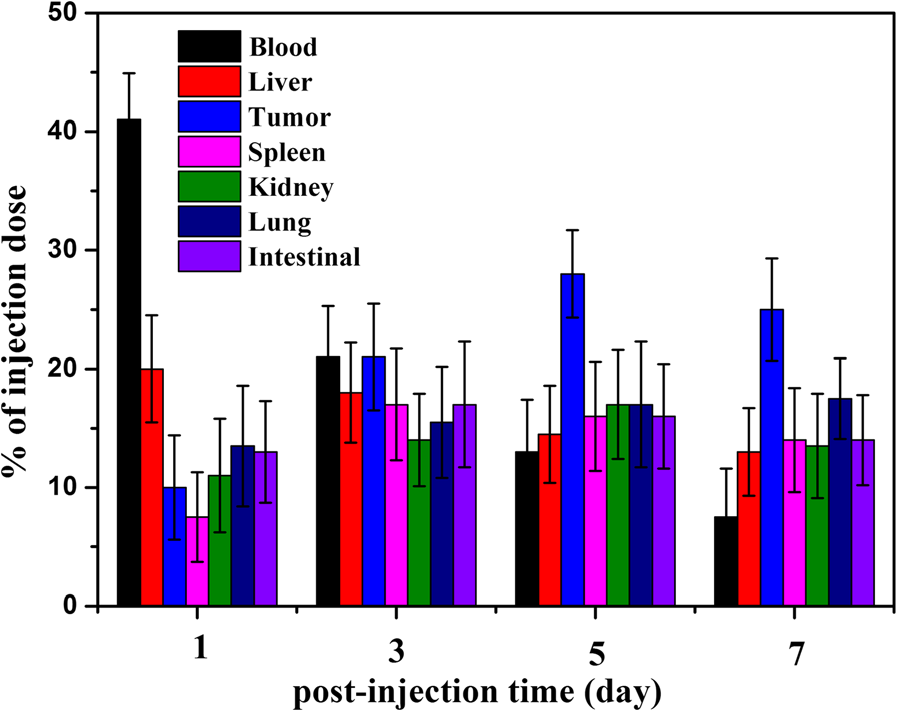
In vivo distribution of CIK cells labeled with Au NRs

As shown in Fig. 8, after NP-CIK cells were injected into gastric cancer-bearing nude mouse models via tail vein injection, at 24 h post-injection, almost 40 % of NP-CIK cells were located in gastric cancer site; as the post-injection day increased, the amount of NP-CIK cells in the tumor site became more and more, at 5 days post-injection, it reached to 28 %, and at 7 days post-injection, it reached to 25 %, which show that NP-CIK cells can target in vivo gastric cancer tissues. In addition, NP-CIK cells also enter into the liver, spleen, kidney, etc.; however, compared with available reports, the amount of gold nanorods loaded with human CIK cells in the tumor site is higher than the amount of antibody-conjugated gold nanorods, which show that human CIK cells should be a good delivery system for multifunctional nanoparticles, therapeutic gene, or drugs.

### CIK Cells Labeled with Au Nanorods for Gastric Cancer Immunotherapy

**Fig. 9:**
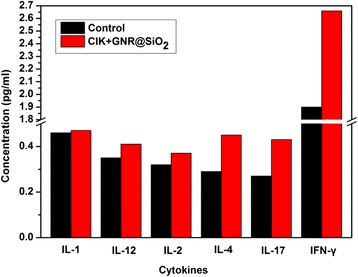
Cytokine concentration at 3 days post-injection of CIK cells labeled with Au nanorods

As shown in Fig. 9, there is no statistical difference in serum levels of cytokines such as IL-1, IL-12, and IL-2 between the experiment groups and control groups at 3 days post-injection, which suggested that no negative inflammatory reaction occurred in experiment groups and the immune safety also was proved unambiguously. Moreover, a statistically distinct increase of serum levels of IL-4, IL-17, and IFN-γ is observed in experiment groups compared with control groups (*P* < 0.05), which indicated that the innate immune was enhanced obviously in experiment groups because of the activation of immune cells, especially to CIK cells, induced by IL-4, IL-17, and IFN-γ.

### CIK Cells Labeled with Au NRs for Photothermal Therapy

**Fig. 10:**
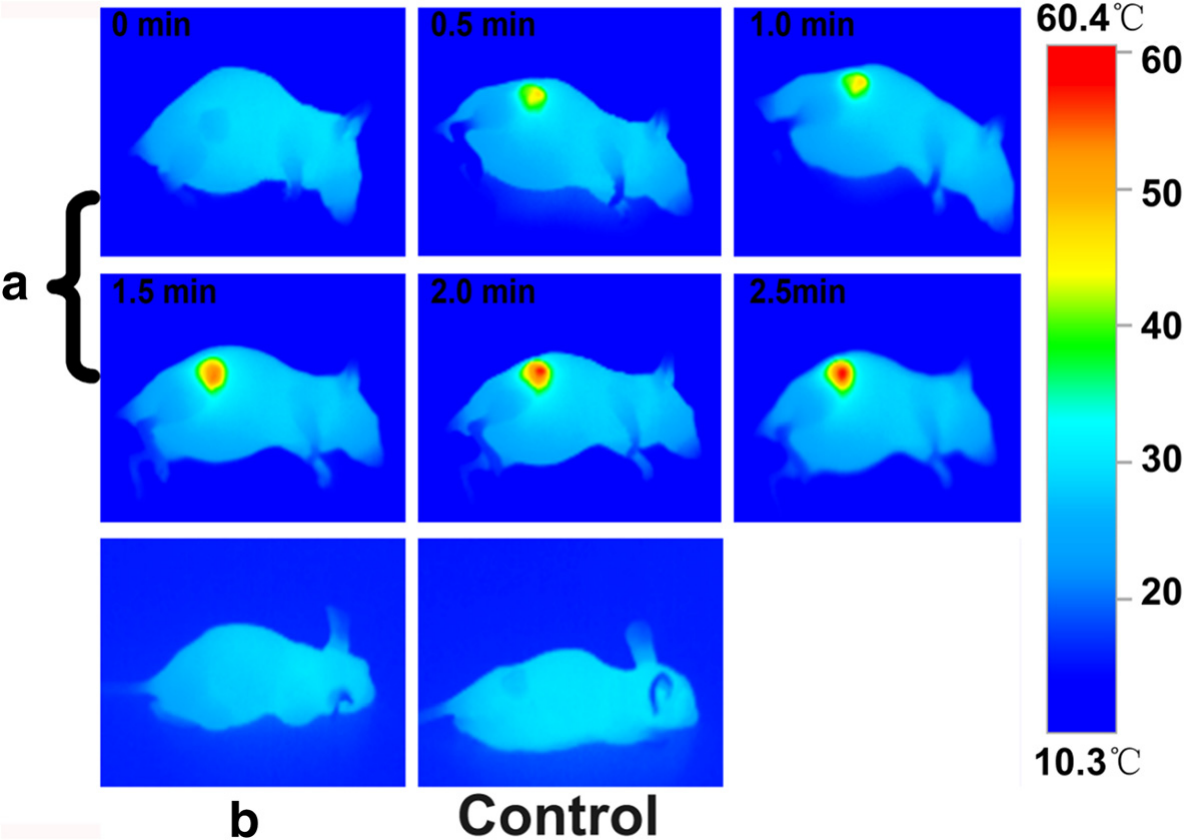
IR images under 1 W/cm^2^ 808-nm NIR laser irradiation. **a** The tumor-bearing mice injected with NP-CIK cells. **b** CIK cell injection group. Control group: 1× PBS injection

As shown in Fig. 10, from the IR images, the NP-CIK cells induced obvious elevation of temperature in the tumor site while no visible changes happened in other groups. According to the bar value, at 1.5 min post-injection, the temperature of the tumor site achieved 57 °C, which is enough to kill tumor cells.

**Fig. 11:**
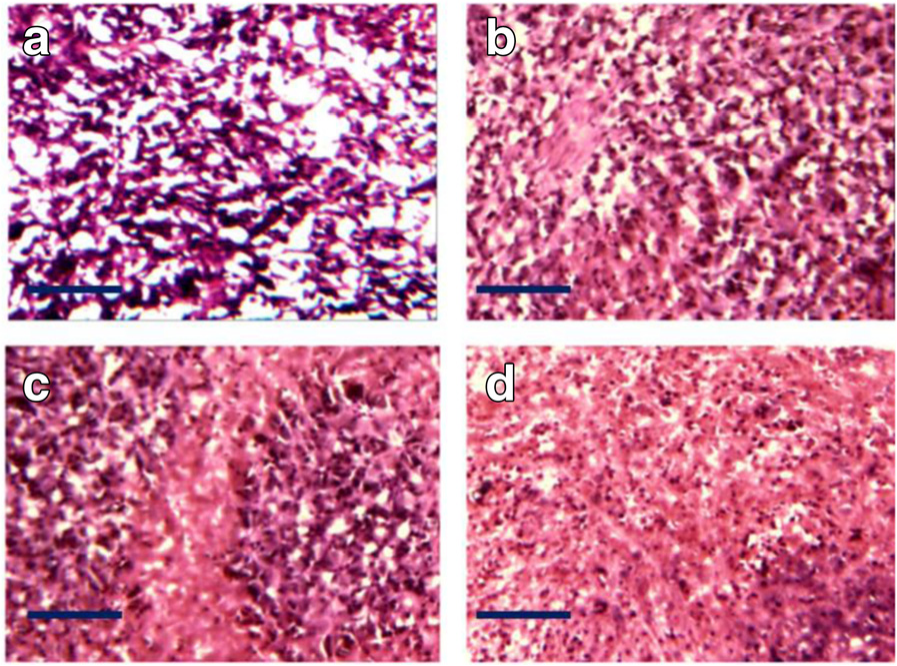
Histological staining of the excised tumors after 808-nm laser irradiation (1.5 W/cm^2^ for 4 min). **a** NP-CIK cell injection with NIR laser irradiation. **b** NP-CIK cell injection without laser irradiation. **c** CIK cell injection with laser irradiation. **d** 1× PBS injection with laser irradiation. *Scale bar* is 10 μm

As shown in Fig. 11, from the histological analysis, the synergistic anti-cancer effects (Fig. 11a) based on NP-based photothermal therapy and CIK cell-based immunotherapy is better than that the effects based on only photothermal therapy (Fig. 11b) or immunotherapy (Fig. 11c). According to this result, CIK cell-based immunotherapy combined with Au NR-based photothermal therapy remarkably enhances the synergistic therapeutic efficacy, which will be a promising treatment for gastric cancer.

**Fig. 12:**
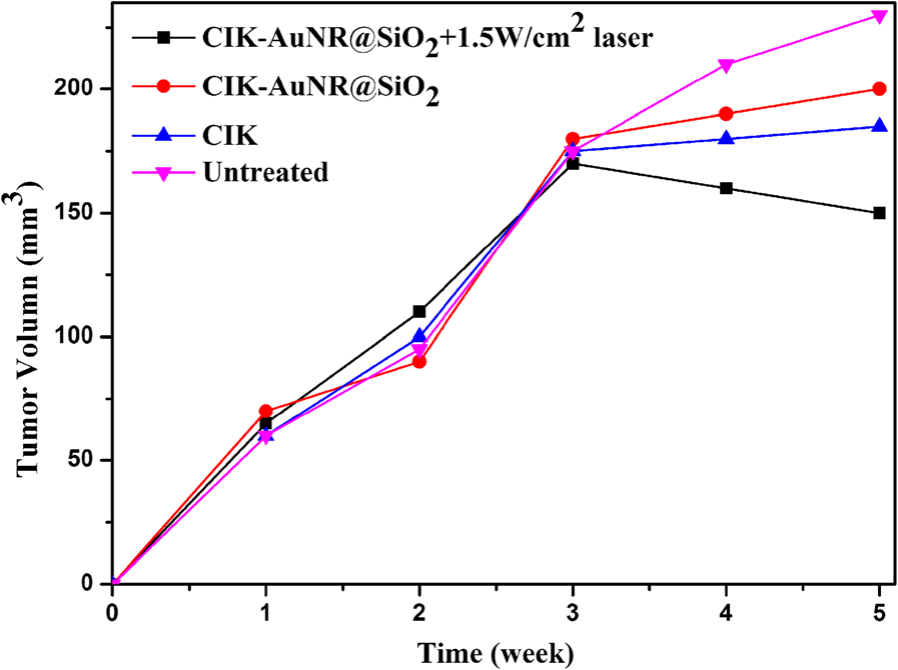
Tumor time-volume curve under different treatment conditions

We also prepared subcutaneous gastric cancer-bearing nude mouse models and raised nude models for 3 weeks. The tumor volume reached to 150–200 mm^3^. When NP-CIK cells were injected into the tumor sites, as shown in Fig. 12, NP-CIK cells + 1.5 W/cm^2^ laser irradiation group obviously inhibited the growth of tumor tissues, which make the tumor size smaller and smaller, compared with only CIK treatment group or Au NR@SiO_2_ + laser irradiation group. There existed statistical difference (*P* < 0.01). NP-CIK cells exhibit better therapeutic effects. Peritumor injection of NP-CIK cells should be a better choice compared with targeted therapy based on tail vein injection and have great potential in applications such as tumor therapy in the near future.

## Conclusions

In summary, we successfully prepared for the first time the CIK cells labeled with Au nanorods and realized targeted gastric cancer fluorescent imaging, photoacoustic imaging, enhanced immunotherapy, and photothermal therapy. Human CIK cells labeled with Au nanorods can target and gather in vivo gastric cancer tissues at 24 h post-injection. As the time post-injection increased, the amount of NP-CIK cells in tumor sites also increased, but there are still NP-CIK cells located in liver, spleen, kidney, and lung organs. Up to date, those NP-CIK cells in liver, spleen, lung, and kidney organs show how to influence their function, which is not still clear, and needs further investigation. As the contrast, NP-CIK cells in tumor site via peritumor injection can stay in tumor sites for a long time, produce better therapeutic efficacy, combined with photothermal therapy of Au nanorods, and exhibit enhanced inhibition of growth of tumor. NP-CIK cells’ peritumor injection may be a better treatment method choice. Due to Au nanorods’ good biocompatibility, therefore, human CIK cells labeled with Au nanorods have their own clinical translation prospect. Furthermore, human CIK cells with Au nanorods designed here are a universal multifunctional theranostic platform [45]. The Au nanorods can be replaced by other nanoparticles with imaging or cancer-killing ability. The human CIK cells in the “CIK-NPs” mode act as a “vehicle,” carrying functional nanoparticles or drugs accurately to the tumor site [46]. Human CIK cells with Au nanorods have great potential in applications such as photoacoustic imaging, immunotherapy, and photothermal therapy of gastric cancer and other tumors in the near future.
